# Effectiveness of intergenerational participation on residents with dementia: A systematic review and meta‐analysis

**DOI:** 10.1002/nop2.919

**Published:** 2021-05-22

**Authors:** Li‐Chin Lu, Shao‐Huan Lan, Yen‐Ping Hsieh, Shou‐Jen Lan

**Affiliations:** ^1^ School of Management Putian University Putian China; ^2^ School of Pharmaceutical Sciences and Medical Technology Putian University Putian China; ^3^ Department of Long‐term Care National Quemoy University Jinning Township Taiwan; ^4^ School of Basic Medical Science Putian University Putian China

**Keywords:** behavioural problem, dementia, intergenerational relations, senile, systematic review

## Abstract

**Aim:**

This meta‐analysis evaluated the effectiveness of intergenerational program participation for long‐term care institution residents with dementia.

**Design:**

A systematic review and meta‐analysis.

**Methods:**

Ten electronic databases were systematically searched until August 2020: CINAHL, the Cochrane Library, EBSCO, EMBASE, Ovid Medline, ProQuest, Psychology and Behavioral Sciences Collection, PubMed, Scopus, and Web of Science. The Joanna Briggs Institute tool (JBI tool) was used for the quality appraisal of the included publications, and Review Manager 5.3 was used for the meta‐analysis.

**Results:**

Thirteen articles were identified (1993–2015). Intergenerational program participation could improve the pleasure level and significantly reduce disengagement behaviours of residents with dementia. The intergenerational program intervention caused no apparent improvement in their quality of life, depression levels, and engagement levels.

## 1 INTRODUCTION

The behavioural and psychological symptoms of dementia (BPSD) reduce social participation and continuous concentration and result in emotional distance and slower reactions in affected patients (Boyle & Malloy, 2004). Recent studies have suggested that social engagement may decrease the risk of dementia and is a modifiable factor. Because loneliness increases the risk of dementia (Aguilera‐Hermida et al., 2018), social engagement can influence individuals' physical and mental conditions through cognitive physical training, thereby delaying or preventing cognitive function decline (James & Bennett, 2019; Qiu & Fratiglioni, 2018; Sakurai et al., 2018).

Intergenerational programs (IPs) are social engagement strategies that can promote cooperation, interaction, and exchange among individuals from two or more generations (Kaplan & Sánchez, 2014). The content of IPs is diverse; they include arts, culture, music, singing, and information and communication technology (Ronzi et al., 2018). Furthermore, IPs can be conducted using the Montessori method (Lee et al., 2007; Sheppard et al., 2016). Participation in IPs provides physical and mental benefits to patients with dementia (Gualano et al., 2018). For example, in some studies, IP participation enabled residents with dementia to become more proactive and happy while reducing their sorrow and anxiety (Baker et al., 2017; Margaret et al., 2014; Mosor et al., 2019; Nyman & Szymczynska, 2016); furthermore, IP participation improved their quality of life (QoL; George, 2011; Su, 2017) and maintained stability of their overall health condition (Margaret et al., 2014). However, limitations still exist; for example, IPs can easily result in the infantilization of participating older adults (Salari, 2002). IP participation has benefits for cognitive and physical functions, and distinguishing these benefits for BPSD can be difficult (Baumgart et al., 2015). Additionally, evidence‐based research on the implementation of IPs is lacking (Middlecamp & Gross, 2002; Salari, 2002). Thus, the effects of IP participation among patients with dementia are unclear (Canedo‐García et al., 2017; James & Bennett, 2019).

In this study, we focused on patients with dementia living in long‐term care institutions (LTC institutions), such as nursing homes, assisted‐living facilities, and day care centres. Daily life in LTC institutions is tedious; the common activities of institution residents include dining and other daily living tasks, and there is a lack of proactive participation in activities (Den Ouden et al., 2015; Smit et al., 2016). Research findings suggested that in LTC institutions, the IP participation of residents without dementia improved their well‐being and communication ability (Blais et al., 2017). Thus, the introduction of IPs in LTC institutions improves residents' daily lives.

Most related studies have verified the benefits of IP participation in patients with dementia through observations or literature reviews (Galbraith et al., 2015; Gualano et al., 2018; Sheppard et al., 2016); few studies have used quantitative meta‐analyses to verify the effectiveness of IP participation. This study focused on studies related to the IP participation of LTC institution residents with dementia and summarized all evidence on the topic to conduct a meta‐analysis. Additionally, we comprehensively discussed the benefits of IP participation for residents with dementia.

## 2 METHODS

### 2.1 Aims

This study assessed the efficacy of IP participation of long‐term care institution residents with dementia through a systematic review and meta‐analysis. The following research question was investigated: What is the efficacy of IP participation of residents with dementia for their QoL, emotions, and behaviours?

### 2.2 Design

The Preferred Reporting Items for Systematic reviews and Meta‐Analyses (PRISMA) guidelines were followed in this study (Moher et al., 2016).

### 2.3 Search strategies and data sources

Studies related to IPs have been conducted in the healthcare field. Thus, the researcher conducted an extensive systematic literature search using 10 online databases until August 26, 2020: CINAHL, the Cochrane Library, EBSCO, EMBASE, Ovid Medline, ProQuest, Psychology and Behavioral Sciences Collection, PubMed, Scopus, and Web of Science. For the literature search, the following keywords were used: “intergenerational program,” “environment,” “dementia,” and “adolescents.” Search terms for IPs were used in combination with terms to identify interventions for dementia‐related keywords. For the search strategies and the search terms, see Supplement 1.

For the inclusion criteria, the PICO (population, intervention, comparison, and outcomes) standard model was used (Eriksen & Frandsen, 2018):

1. Population: Participants should be older individuals with dementia.
2. Intervention: IPs should be adopted, which were defined as activities with the interaction of older and younger people.
3. Comparison: The comparison group included older individuals with dementia receiving routine care.
4. Outcomes: QoL, depression, disengagement, and engagement level were the outcomes studied.

### 2.4 Study types

This study discussed the efficacy of IP interventions for dementia patients. Thus, studies with a causal design were included, including comparative studies, clinical trials, and randomized control trials (RCTs) with cross‐sectional, case–control, prospective, and retrospective designs.

### 2.5 Inclusion and exclusion criteria

This study reviewed all titles and abstracts from the retrieved articles. The articles were selected based on the following inclusion criteria: (a) study types: comparative studies, clinical trials, and RCTs with cross‐sectional, case–control, prospective, and retrospective designs; (b) study participants: dementia patients who interacted with children, youth, or adolescents; (c) participants' data and at least one mental health outcome were provided; and (d) articles published in English.

Articles were excluded based on the following exclusion criteria: (a) review articles, editorials, books, letters, case reports, posters, news, or comments; (b) duplicate articles or results; (c) study participants were hospitalized; (d) articles included nonhuman or infant participants; and (e) non‐full‐text online articles.

### 2.6 Quality appraisal of publications

Three investigators independently reviewed the extracted data on methodologies, results, study populations, intervention types, and potential sources of bias. In this study, the Joanna Briggs Institute tool (JBI tool; Peters et al., 2015) was used for the assessment of publication quality. The JBI publishes assessment item lists according to research types (Hannes & Lockwood, 2011). Publications that satisfied over 50% of the JBI's assessment item lists for different types of research methods were considered to have high quality.

For RCTs, the JBI lists have 13 assessment items, and six of them must be achieved. For prospective retrospective studies, the lists have nine assessment items, and five must be achieved. For cohort studies, the lists have 11 assessment items, and six must be achieved. For qualitative studies, the lists have 10 assessment items, and five must be achieved. The aforementioned criteria must be achieved for classifying the publications as having high quality.

A total of 13 studies were classified as having high quality according to the JBI criteria (Supplement 2). The article by Jarrott and Bruno (2007) was regarded to be high risk, while it did not run across more than half of the overall grade and supposing that it was with manifest defects. Disagreements were resolved by referral to or discussion with a superior investigator to attain a consensus. After such a discussion, the study by Jarrott and Bruno (2007) was included.

### 2.7 Data extraction

The study selection criteria were as follows: (a) comparative results were provided; (b) participants were adults or elders residing in care institutions, nursing homes, or day care centres; (c) the interventions were IPs; (d) the study investigated BPSD, engagement levels, and QoL; and (e) weighted mean difference (WMD) or dichotomous outcomes with sufficient data were available.

### 2.8 Synthesis

Mean differences (MDs) and corresponding 95% confidence intervals (CIs) were calculated for each study outcome (e.g., BPSD [engagement levels], depression, and QoL). IP participants were considered the “intervention group,” whereas the other group that did not interact with younger individuals or children was considered the “control group.” A meta‐analysis was performed on the outcomes of QoL, depression measures, and engagement levels (levels of pleasure, self‐engagement [SE], active engagement [AE], passive engagement [PE], and disengagement) using the Review Manager software package (RevMan, 5.3; Cochrane's Informatics & Knowledge Management Department). The heterogeneity of intergenerational care effects between studies was evaluated using the *Q* (heterogeneity *X* ^2^) and *I* ^2^ statistics (Higgins & Thompson, 2002; Higgins et al., 2003). The random‐effects model was applied when the *Q* statistics were PQ < 0.1 or *I* ^2^ > 50%, suggesting the presence of heterogeneity (DerSimonian & Kacker, 2007); otherwise; when the *Q* statistics were PQ > 0.1 and *I* ^2^ < 50% (suggesting the absence of heterogeneity), the fixed‐effects model was applied (Mantel & Haenszel, 1959). Overall effects were determined using the *Z*‐test.

## 3 RESULTS

### 3.1 Study characteristics and study quality

The search program generated 1,248 articles; 453 articles were excluded because of duplication, and 779 articles were considered irrelevant based on the title and abstract; 16 full‐text articles were retrieved for examination: three articles were considered irrelevant based on the full text; thus, the remaining 13 articles were included in the final review. Finally, six studies were included in the meta‐analysis based on the inclusion and exclusion criteria (Supplement 3).

Thirteen intergenerational care studies including one qualitative study (Lokon et al., 2012), one crossover study (Ward et al., 1996), three RCTs (George, 2011; Lee et al., 2007; Low et al., 2015), four pretest–posttest studies (Kamei et al., 2011; Skropeta et al., 2014; Camp et al., 1997; Xaverius & Mathews, 2004), and four prospective studies (Jarrott & Bruno, 2003, 2007; Newman & Ward, 1993; Sauer et al., 2014) published between 1993–2015 met the inclusion criteria. Of these studies, 10 were conducted in the USA, two were in Australia, and one was in Japan.

The characteristics of participants including dementia patients and children (e.g., by facility, age, and group) varied across the studies. The 13 studies included participants with dementia; children and youth participants included preschool children, elementary school students, and university students. IP outcomes related to the interaction effect included mental health and engagement measures. The study quality and characteristics are detailed in Table 1.

**TABLE 1:** Characteristics and results of studies included in the review

Author, publication year Country	Study design,	Duration of intervention	Facility	(Ageing participants, Age)	(Child participants, Age)	Study site Environmental considerations implemented	Outcomes	JBI qualities
Newma, 1993 USA	Prospective controlled clinical trail	5 weeks	Council Care Senior Adult Day Care Centers	Older adults with dementia (*N* = 21) (Age: 50–90 years)	Preschoolers children (7–8 grade, *N* = 11; 8–9 years, *N* = 9)	Adult day care centres Wednesday, morning	Significant increase with the children present: touching (*p* = .051) and extending hands (*p* = .04)	High
Ward, 1996 USA	Cross over design	6 months	Metropolitan Jewish Geriatric Center	person with dementia (*N* = 21) (Mean age 85)	Children (*N* = 24) (age: Unclear)	Metropolitan Jewish Geriatric Center (nursing facility‐359‐bed)	1. Touching was more frequent 2. Residents' agitation levels decreased	High
Camp, 1997 USA	Before and after design pilot study	75 weeks	long‐term care facilities	Older adults with dementia (*N* = 12) (age: 70–96 years)	Children (*N* = 14) (age: 2.5–5 years)	Small dining room on a regular schedule, in the same settings and circumstances, (e.g., once a week (Tuesdays) from 9:45 a.m. until 10:15 a.m.)	1. Older adults could indeed serve as mentors and teachers in this intergenerational program 2. No becoming aggressive, disruptive, confused, or anxious during any activity sessions with children	High
Jarrott, 2003 USA	Prospective controlled clinical trail	23 months	ONE generation Daycare clients	Older adults with dementia (*N* = 48) (Mean age79) Control group (*N* = 27) Intervention (*N* = 21) (self‐selected)	Children (infants from 6 weeks–12 months, toddlers between 13–24 months, 2–3 year olds, 3–4 year olds, or 4–5 year olds)	ONE generation Daycare	1. MMSE was not associated with affect (*p* > .1) 2. Treatment group members attended the program more days per week than comparison group participants	High
Xaverius, 2004 USA	Before and after design (one group repeat measure)	18 weeks	Study 2: Special care unit	Older adults with dementia (*N* = 25) (age: 75–98 years)	Children (*N* = 60)(second‐graders) (age: Unclear) Control group (*N* = 32) Intervention (*N* = 28)	Friday, morning (11:00‐noon) The lounge areas had several tables, each with four chairs around them and a television, sofa, and lounge chair in the corner of the room. In another corner of the room was a nurse's station	More engaged (*t*(85) = 4.60, *p* > .001) and expressive (*t*(85) = 5.55, *p* > .001) in the treatment condition than the control condition	High
Jarrott, 2007 USA	Prospective controlled clinical trail	5 years (average)	ONE generation Daycare clients	Older adults with dementia (*N* = 78) (Mean age 80)	Children (*N* = Unclear) (age: 6 weeks–6 years)	ONE generation Daycare semiprivate room	1. 97% (*N* = 38) indicated, they benefited from the IG programming 2. Benefits included appreciation for diversity, formation of close intergenerational relationships, and enhanced client self‐esteem	High
Lee, 2007 USA	Randomized control trial	6 months/per group	A dementia special care unit at a skilled nursing facility	Older adults with dementia (*N* = 14) (Mean age 90.29) Control group (*N* = 7) Intervention (*N* = 7)	Children (*N* = 15) (2–5 dyads) (age: 2.5–5 years)	Specific nursing	More constructive engagement (*t*(13) = 22.90, *p* ≤ .001), less passive engagement (*t*(13) = 6.55, *p* ≤ .001), less active engagement (*t*(13) = 6.62, *p* < .001), less self‐engagement (*t*(13) = 4.70, *p* ≤ .001 and less no engagement (*t*(13) = 5.56, *p* ≤ .001)compared to that of regular unit activities	High
George, 2011 USA	Randomized control trial	5 months	Northeast Ohio community and local assisted living and nursing homes	Older adults with dementia who scoring above a 5 on the MMSE (*N* = 15) (Mean age 83.55) Control group (*N* = 7) Intervention (*N* = 8)	Children (*N* = 16) (age: 5–6 years and 11–14 years)	Local assisted living and nursing homes	1. Decrease in stress for the intervention group 2. Quality of life (QOL): perceived health benefits, sense of purpose and sense of usefulness, and relationships	High
Kamei, 2011 Japan	Before and after design	6 months	Obtained space in the college building	Older people with slight dementia Intergenerational day program (IDP) group (*N* = 14) (Mean age 75.6) (Program volunteers) (*N* = 8) (Mean age 68.6) Control group: two groups' baseline	Elementary‐school children (*N* = 7)(age: Unclear)	Multipurpose room (6.7 * 11.9 m) of nursing home 2:00–5:00 p.m.	1. The quality of life (HRQOL) on mental health (*p* = .03) improved significantly between the first involvement and after 6 months 2. GDS‐15 scores (*p* = .045) significantly decreased at the three time points	High
Lokon, 2012 USA	Qualitative study	10–12 weeks	Long‐term care facilities	Older people with dementia (*N* = approximately 150 pairs) (Mean age Unclear)	College Students (*N* = approximately 150 pairs) (Mean age Unclear)	Public gallery exhibition	Maintained greater functional Stay indoors more often Physical activity is unclear	High
Sauer, 2014 USA	Prospective controlled clinical trail	15 months	Long‐term care facilities	Older people with dementia (age: Unclear) Opening Minds through Art (OMA) (*N* = 38) Traditional visual arts activities (*N* = 10)(observed biweekly)	College Students	Public gallery exhibition long‐term care facilities and adult day centres	1. Well‐being (i.e. social interest, engagement, and pleasure) mean intensity score was significantly higher during OMA 2. Not significant difference in the total combined ill‐being (i.e. disengagement, negative affect, sadness, and confusion)	High
Skropeta, 2014 Australia	mixed methods quantitative and qualitative design	6 months	Uniting Care Ageing facilities	Older people with cognitively intact and impaired (*N* = 48) (Mean age 85)	Children (*N* = 50) (age: 0–4 years)	Hostel, nursing home, specific nursing home	Older adults: SF36 pretest and posttest results indicated a decrease on Scale 4 – Energy/fatigue *F* (1, 31) = 10.957. *p* = .002 Geriatric Depression Scale (GDS) showed no significant differences	High
Low, 2015 Australia	Randomized control trial	3 months	Residential aged‐care facility	Residents with cognitive impairment (80%) (*N* = 40) (aged: over 65 years) Control group (*N* = 20) Grandfriends participants (*N* = 20)	Preschool children (*N* = 21) (age: 3–5 years)	Residential aged‐care facility on a large site	1. Increased passive engagement and enjoyment among nursing home residents when interacting with preschoolers 2. Quality of life, agitation, brief sense of community did not show changes	High

### 3.2 Efficacy on QoL

**FIGURE 1:**
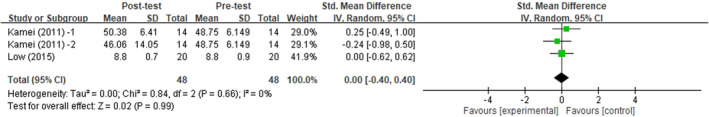
Forest plot of the quality of life (QOL)

Participants with dementia who received intergenerational care program intervention exhibited no significant difference in QoL. Two studies assessed QoL, with 34 participants in the intervention group (Kamei et al., 2011; Low et al., 2015). The pooled results indicated that the intergenerational care program intervention received had no influence on QoL, with a standardized mean difference (SMD) of 0.00 (%) (95% CI: −0.40 to 0.40, *p* = .99; *I* ^2^ = 0%) (Figure 1).

### 3.3 Efficacy on depression

**FIGURE 2:**
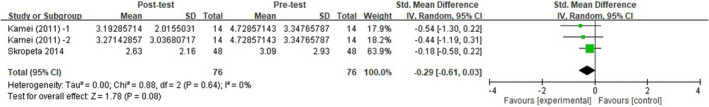
Forest plot of the depression

Two studies with a pretest–posttest design assessed depression in 62 participants (Kamei et al., 2011; Skropeta et al., 2014). The pooled results revealed that the received intergenerational care program intervention did not ameliorate patients' depression, with an SMD of −0.29% (95% CI: −0.61 to 0.03, *p* = .08; *I* ^2^ = 0%) (Figure 2).

### 3.4 Efficacy on engagement

**FIGURE 3:**
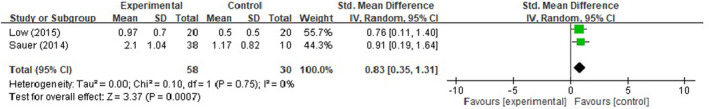
Forest plot of the pleasure

Two studies assessed pleasure levels, with 58 participants in the IP group and 30 in the control group (Low et al., 2015; Sauer et al., 2014). The pooled results indicated significantly increased pleasure levels in the intervention group, with an SMD of 0.83% (95% CI: 0.35 to 1.31, *p* = .0007; *I* ^2^ = 0%) (Figure 3).

**FIGURE 4:**
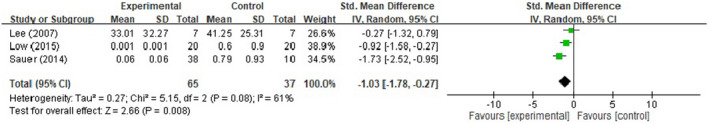
Forest plot of the disengagement

Three studies assessed disengagement levels, with 65 participants in the intervention group and 37 in the control group (Lee et al., 2007; Low et al., 2015; Sauer et al., 2014). The pooled results indicated that the received intergenerational care program intervention decreased disengagement levels in the intervention group, with an SMD of −1.03% (95% CI: −1.78 to −027, *p* = .008; *I* ^2^ = 61%) (Figure 4).

**FIGURE 5:**
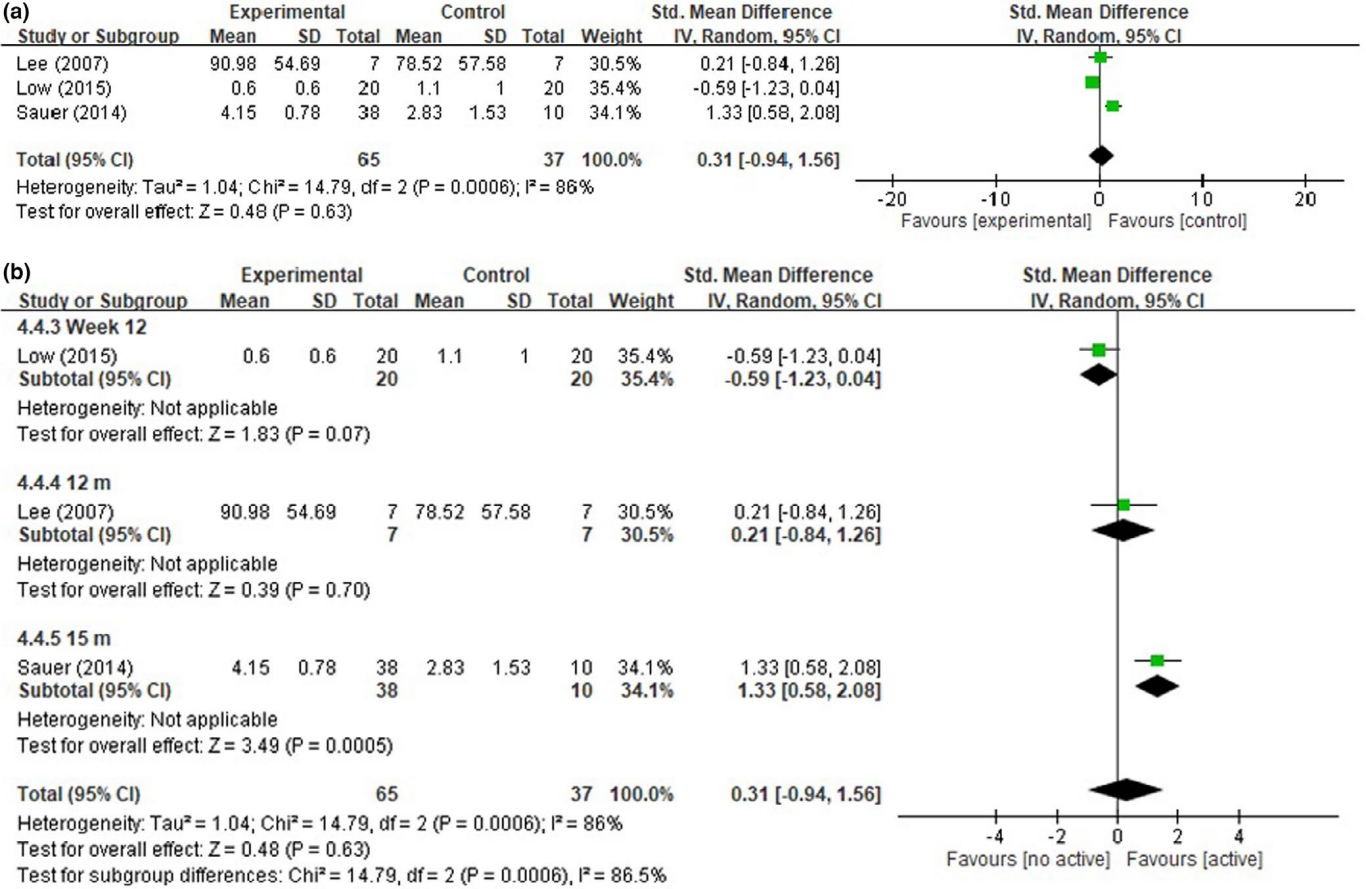
(a) Forest plot of the active engagement (AE). (b) Forest plot of the active engagement (AE) by endpoint time

Three studies compared AE levels, with 65 participants in the intervention group and 37 in the control group (Lee et al., 2007; Low et al., 2015; Sauer et al., 2014). The pooled results revealed no significant difference in AE levels, with an SMD of 0.31% (95% CI: −0.94 to 1.56, *p* = .63; *I* ^2^ = 86%) (Figure 5a). No significant difference in AE levels was found in an analysis stratified by endpoint time (Figure 5b).

**FIGURE 6:**
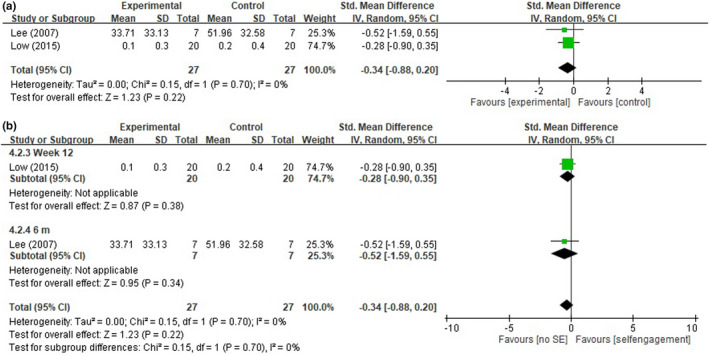
(a) Forest plot of the self‐engagement (SE). (b) Forest plot of the self‐engagement (SE) by endpoint time

Two studies compared SE levels, with 27 participants in the intervention group and 27 in the control group (Lee et al., 2007; Low et al., 2015). The pooled results revealed that the received intergenerational care program intervention might not have significant effects on the SE level, with an SMD of −0.34% (95% CI: −0.88 to 0.20, *p* = .22; *I* ^2^ = 0%) (Figure 6a). No significant difference was found in SE levels in the analysis stratified by endpoint time (Figure 6b).

**FIGURE 7:**
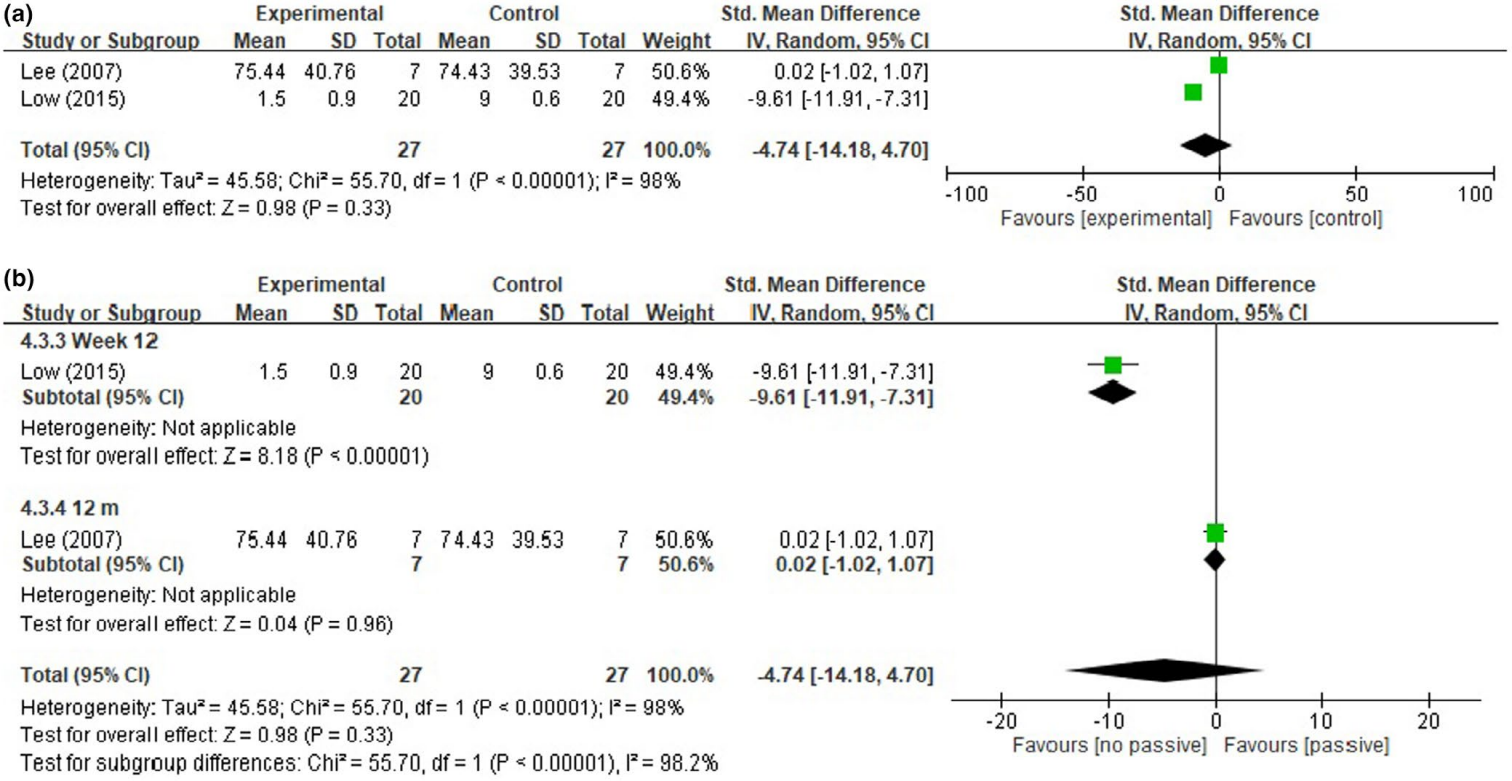
(a) Forest plot of the passive engagement (PE). (b) Forest plot of the passive engagement (PE) by endpoint time

Two studies compared PE levels, with 27 participants in the intervention group and 27 in the control group (Lee et al., 2007; Low et al., 2015). The pooled results indicated that the received intergenerational care program intervention did not have significant effects on PE levels, with an SMD of −4.74 (%) (95% CI: −14.18 to 4.70, *p* = .33; *I* ^2^ = 98%) (Figure 7a). No significant difference was found in PE levels in the analysis stratified by endpoint time (Figure 7b).

No statistically significant heterogeneity was found in studies reporting SE levels (*I* ^2^ = 0%); heterogeneity was found between studies examining pleasure levels, disengagement levels, AE levels, and PE levels (*I* ^2^ = 61%–98%).

## 4 DISCUSSION

Our meta‐analysis results suggested that IP participation significantly increased pleasure levels and reduced behavioural disengagement among residents with dementia but did not significantly improve QoL and depression, AE, SE, and PE levels.

Our findings indicated that IP participation increased pleasure levels and reduced behavioural disengagement, such as zoning out, sleeping, and leaving the activity zone, in patients with dementia (Lee et al., 2007; Low et al., 2015; Sauer et al., 2016). Two factors potentially affected the happy mood of patients with dementia who participated in IPs. First, decreases in working memory and executive function in patients with dementia are correlated with their disengagement of attention (Crawford et al., 2015). Patients with Alzheimer's disease have slower visual responses to precautions (Crawford et al., 2015). Therefore, studies have recommended that using cues containing exogenous components can be more effective (Tales et al., 2002). Well‐trained staff members play a pivotal leadership role in IPs. Additionally, individuals from different generations are encouraged and guided to establish an interactive relationship with residents with dementia (Bethany et al., 2015; Driessen, 2018; Low et al., 2015; Perugia et al., 2018).

Second, studies have stated that for residents with dementia, one‐on‐one live human interaction is the most crucial factor influencing their pleasure (Cohen‐Mansfield et al., 2012), which can have a substantial effect on their attention and executive functions. This effect also enables dementia patients to participate for longer in activities. Moreover, appropriate cues provided by well‐trained staff members contribute to a reduction in patients' behavioural disengagement (Kolanowski et al., 2012; Van Haitsma et al., 2013).

Our findings indicated that residents with dementia who participated in IPs did not have a significant improvement in their QoL; QoL is a multilayered concept related to physiological, psychological, social, and emotional levels. This study incorporated QoL dimensions from two studies. Kamei et al. (2011) used eight QoL dimensions of the Japanese version of SF‐8 (Fukuhara & Suzukamo, 2004), and QoL was improved following the research intervention. Low et al. (2015) used the five dimensions of the long‐term care QoL scale in a related study (McDonald, 2013). Short‐term interventions cannot be used to evaluate various QoL dimensions. Future studies must focus on the relationship between each QoL dimension of residents with dementia and their IP participation.

IP interventions could not improve the depression status of residents with dementia. Depression and dementia are comorbidities, and a correlation between depression and a decrease in the volume of the hippocampus and entorhinal cortex was found in anatomy and nuclear medicine research (O'Shea et al., 2018). Additionally, patients with dementia mostly take antipsychotics and antidepressants as medication for dementia (Ford et al., 2018); medical treatment is required for their depression. Research suggests that increasing patients' activities can alleviate their depression symptoms (Poelke et al., 2016). However, the two studies mentioned in this study suggest a decreasing trend of depression symptoms in residents with dementia, but the decrease was nonsignificant.

AE, SE, and PE levels among patients with dementia were not significantly different after they participated in IPs. However, due to the small sample size and high heterogeneity between AE and PE outcomes, the findings should be interpreted with caution. The reason for this high heterogeneity is the different assessment tools. For example, Low et al. (2015) conducted observation and classification on highly experienced researchers; Sauer et al. (2014) used the revised Greater Cincinnati Chapter Well‐Being Observation Tool (GCWBT) to code participants' behaviour.

The following factors are proposed as the possible causes for the lack of evidence for IP participation changing the behaviour of patients with dementia. First, a low level of participation in activities is correlated with neurobiological changes. Medical diagnoses suggest that left‐lateralized salience network dysfunction in patients with behavioural variant frontotemporal dementia (bvFTD) affects the activity of the parasympathetic nervous system, leading to antisocial behaviours (Sturm et al., 2018). Therefore, those with bvFTD are not adept at adjusting their reactions to their environment and at sharing with, recognizing, and responding to others (Hua et al., 2018). Moreover, depression is one of the factors for patients' low participation in social activities due to its high correlation with dementia (Kim et al., 2016). Third, dementia‐related diseases lead to the intercorrelation of changes in cognition, memory, identity, and depressive moods. Qualitative research indicates that patients with dementia have difficulty deriving pleasure from social activities due to changes in identity and self‐awareness (Caddell & Clare, 2011). This suggests that the participation of LTC institution residents with dementia in IPs may need to be examined from multiple perspectives.

Research suggests that IPs have protective effects on hippocampal atrophy and can improve cognitive performance, indicating that social participation is effective at improving neuroplasticity (Sakurai et al., 2018). Although we were unable to verify the benefits of participating in IPs for residents with dementia, this study did confirm that participation improved pleasure levels and reduced behavioural disengagement.

This study has several limitations. First, the studies analysed rarely categorized dementia according to disease severity and type. Patients with different types and severity levels of dementia might have engaged in IPs using different methods and might have had different participatory conditions (Rasmussen et al., 2019). Consequently, this study failed to verify dementia patients' participatory conditions in IPs. Furthermore, in IP‐related research, cognitive function, medication, and medical care have rarely been discussed. Therefore, we could not verify the effect of IP participation of patients with dementia on their disease status. This highlights the need to incorporate more issues related to medical care for dementia in IP‐related research.

## 5 CONCLUSION

The results suggest that residents with dementia who participated in IPs had increased pleasure levels and reduced behavioural disengagement. We were unable to confirm the correlations among the QoL, depression, and participatory conditions of residents with dementia. However, research reports have indicated that engaging in pleasurable activities at institutions can reduce the medication usage of patients. Therefore, future research should explore neural mechanisms related to IPs in institution residents with dementia receiving medical care.

## CONFLICT OF INTEREST

The authors declare no conflicts of interest.

## AUTHOR CONTRIBUTIONS

YPH: Study design. YPH and LCL: Planning, coordination, and management of data acquisition. LCL and YPH wrote the first draft of the manuscript. LCL and SHL wrote the revision of the manuscript. LCL, SHL, and SJL have contributed to the statistical planning of the study. All authors participated in the critical revision of the manuscript and gave final approval of the article.

## ETHICAL APPROVAL

Not applicable.

## Supporting information

Supplementary Material

## Data Availability

The data used to support the findings of this study are available from the corresponding author upon request.
