# Supplementary material for: Effectiveness of intergenerational participation on residents with dementia: A systematic review and meta‐analysis
Source: Nurs Open. 2021 May 22;9(2):920–31. doi: 10.1002/nop2.919 (PMC8859040; doi:10.1002/nop2.919)
Supplement: Supplementary file 1 — Supplementary Material [file NOP2-9-920-s001.docx]

Supplement 1. Search Terms Used for All Databases

| Search terms | Search strategies |
| --- | --- |
| intergeneration program | Inter-generat* OR intergenerat* OR cross-generational OR cross-age OR multi-generation OR multigenerat* OR trans-generation* OR transgenerat* OR age integration |
| environment | Environment* |
| dementia | Dementia OR alzheimer* OR cogni* OR alzh* |
| adolescents | Adolescent* OR child* OR student* OR youth* OR volunteer* OR teens |

| Supplement 2. Present results of JBI quality across studies | | | | |  |  |  |  |  |  |  |
| --- | --- | --- | --- | --- | --- | --- | --- | --- | --- | --- | --- |
| **RCT Studies** | Was true randomization used for assignment of participants to treatment groups? | Was allocation to treatment groups concealed? | Were treatment groups similar at the baseline? | Were participants blind to treatment assignment? | Were those delivering treatment blind to treatment assignment? | Were outcomes assessors blind to treatment assignment? | Were treatments groups treated identically other than the intervention of interest? | Was follow-up complete, and if not, were strategies to address incomplete follow-up utilized? | Were participants analysed in the groups to which they were randomized? | Were outcomes measured in the same way for treatment groups? | Were outcomes measured in a reliable way? |
| Lee 2007 | 🗸 | unclear | 🗸 | 🗸 | unclear | 🗸 | 🗸 | low risk | 🗸 | 🗸 | 🗸 |
| Low 2015 | 🗸 | 🗸 | 🗸 | 🗸 | 🗸 | 🗸 | 🗸 | 🗸 | 🗸 | 🗸 | 🗸 |
| George 2011 | 🗸 | 🗸 | 🗸 | 🗸 | 🗸 | 🗸 | 🗸 | 🗸 | 🗸 | 🗸 | 🗸 |
| **Prospective retrospective Studies** | Is it clear in the study what is the ‘cause’ and what is the ‘effect’ (i.e. there is no confusion about which variable comes first)? | Were the participants included in any comparisons similar? | Were the participants included in any comparisons receiving similar treatment/care, other than the exposure or intervention of interest? | Was there a control group? | Were there multiple measurements of the outcome both pre and post the intervention/exposure? | Was follow-up complete, and if not, was follow-up adequately reported and strategies to deal with loss to follow-up employed? | Were the outcomes of participants included in any comparisons measured in the same way? | Were outcomes measured in a reliable way | Was appropriate statistical analysis used? |  |  |
| Ward 1996 | 🗸 | 🗸 | 🗸 | 🗸 | unclear | low risk | 🗸 | 🗸 | 🗸 |  |  |
| Newma 1993 | 🗸 | 🗸 | 🗸 | 🗸 | 🗸 | low risk | 🗸 | 🗸 | 🗸 |  |  |
| Sauer 2014 | 🗸 | 🗸 | 🗸 | 🗸 | unclear | low risk | 🗸 | 🗸 | 🗸 |  |  |
| Jarrott 2007 | 🗸 | 🗸 | unclear | unclear | unclear | low risk | 🗸 | unclear | 🗸 |  |  |
| Jarrott 2003 | 🗸 | 🗸 | 🗸 | 🗸 | unclear | low risk | 🗸 | 🗸 | 🗸 |  |  |
| **Cohort Studies** | Were the groups similar and recruited from the same population? | Were the exposures measured similarly to assign people | Was the exposure measured in a valid and reliable way? | Were confounding factors identified? | Were strategies to deal with confounding factors stated? | Were the groups/participants free of the outcome at the start of the study (or at the moment of exposure)? | Were the outcomes measured in a valid and reliable way? | Was the follow up time reported and sufficient to belong enough for outcomes to occur? | Was follow-up complete, and if not, were the reasons to loss to follow-up described and explored? | Were strategies to address incomplete follow up utilized? | Was appropriate statistical analysis used? |
|  |  | to both exposed and unexposed groups? |  |  |  |  |  |  |  |  |  |
| Kamei 2011 | 🗸 | 🗸 | 🗸 | 🗸 | unclear | 🗸 | 🗸 | 🗸 | low risk | low risk | 🗸 |
| Skropeta 2014 | 🗸 | 🗸 | 🗸 | unclear | unclear | 🗸 | 🗸 | 🗸 | low risk | low risk | 🗸 |
| Xaverius 2004 | 🗸 | 🗸 | 🗸 | unclear | unclear | 🗸 | 🗸 | 🗸 | low risk | low risk | 🗸 |
| Camp 1997 | 🗸 | 🗸 | 🗸 | unclear | unclear | unclear | 🗸 | 🗸 | low risk | low risk | 🗸 |
| **Qualitative Studies** | Is there congruity between the stated philosophical | Is there congruity between the research methodology | Is there congruity between the research methodology | Is there congruity between the research methodology | Is there congruity between the research methodology | Is there a statement locating the researcher culturally | Is the influence of the researcher on the research, and | Are participants, and their voices, adequately | Is the research ethical according to current criteria or, | Do the conclusions drawn in the research report flow |  |
|  | perspective and the research methodology? | and the research question or objectives? | and the methods used to collect data? | and the representation and analysis of data? | and the interpretation of results? | or theoretically? | vice- versa, addressed? | represented? | for recent studies, and is there evidence of ethical | from the analysis, or interpretation, of the data? |  |
|  |  |  |  |  |  |  |  |  | approval by an appropriate body? |  |  |
| Lokon 2012 | 🗸 | 🗸 | 🗸 | 🗸 | 🗸 | 🗸 | unclear | 🗸 | 🗸 | 🗸 |  |


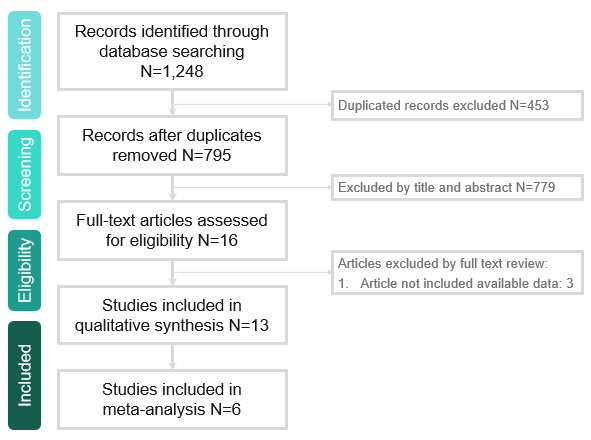


Supplement 3. Flowchart of search results and article retrieval

The electronic database searches found a total of 1,248 records, and of this total number 16 full texts were retrieved for detailed examination. A total of 13 articles were included in the final review and 6 articles included in meta-analysis. The main reasons were that the studies not meet inclusion criteria.
